# The Association Between Inflammatory Scores and Frailty Severity: An Exploratory Retrospective Analysis in Non-Small-Cell Lung Cancer Surgical Patients

**DOI:** 10.3390/medsci14020170

**Published:** 2026-03-28

**Authors:** Radu-Alexandru Iacobescu, Vasile Lucian Boiculese, Gianina Dodi, Tiberiu Lunguleac, Cristina Grigorescu, Sabina Antoniu

**Affiliations:** Grigore T. Popa University of Medicine and Pharmacy Iasi, 700115 Iași, Romania; lboiculese@gmail.com (V.L.B.); gianina.dodi@umfiasi.ro (G.D.); tiberiu.lunguleac1@umfiasi.ro (T.L.); cristina.grigorescu@umfiasi.ro (C.G.); sabina.antoniu@outlook.com (S.A.)

**Keywords:** biomarkers, comorbidities, frailty, inflammation, lung cancer, non-small-cell lung cancer surgery

## Abstract

Background: Frailty has been linked with systemic inflammation in elderly oncology patients. In this paper, we report the results of an analysis evaluating the association between blood cell biomarkers of inflammation and frailty in patients with operable non-small-cell lung cancer (NSCLC). Methods: A retrospective analysis was performed on patients undergoing surgery for NSCLC between March 2022 and March 2023. Frailty was assessed using the modified Frailty Index-5 (mFI-5) and 11 (mFI-11). Inflammation was evaluated using the neutrophil-to-lymphocyte ratio (NLR), platelet-to-lymphocyte ratio (PLR), monocyte-to-lymphocyte ratio (MLR), systemic immune–inflammation index (SII), and systemic immune–inflammation response index (SIRI), all calculated from preoperative assessments. Results: In this sample of surgical NSCLC patients (n = 106), frailty prevalence was 29.2% with mFI-11 and 17% with mFI-5. The log of the neutrophil-to-lymphocyte ratio (logNLR) emerged as the most significant predictor of frailty (OR of 3.13, 95% CI: 1.12–9.09, *p* = 0.03 for mFI-11 and 3.82, 95% CI: 1.28–11.11, *p* = 0.02 for FI-5). The platelet-to-lymphocyte ratio (PLR) was predictive only in the model assessing mFI-5. Furthermore, both the NLR and PLR showed an inverse linear correlation with frailty severity, a finding that remained consistent regardless of age, gender, disease stage, nutrition status, or comorbidity burden. Conclusions: Frail patients with operable NSCLC exhibit distinct inflammatory response patterns compared with those observed in non-frail lung cancer patients. Using these biomarkers could help identify patients suitable for preoperative interventions that could improve their postoperative course. However, further studies are needed to explore these preliminary findings prospectively and to understand the causal relationship between the observed association with frailty status and severity.

## 1. Introduction

Lung cancer is a prevalent condition and is predominantly considered an age-related disease, as most patients are above the age of 60 (mean age at diagnosis of 71) [1,2]. These patients often exhibit higher frailty levels than the general population, estimated at 12% when assessed with frailty-phenotype-derived screening tools and up to 24% when assessed with deficit accumulation models [3]. Some estimates suggest that up to half of lung cancer patients are frail at diagnosis [4].

Several factors are said to contribute to the increased frailty burden in patients with cancer, including nutrition, age, and comorbidity [5]. Chronic inflammation was reported to be associated with decreased physical function and with increased disease risk in many clinical settings, including lung cancer [6,7]. Furthermore, chronic inflammation can result in frailty, and this relationship is the most obvious in the case of aging, with inflammaging being demonstrated to be associated with frailty [8]. In fact, frail patients have been shown to exhibit higher levels of chronic inflammation biomarkers such as CRP (C-reactive protein) and IL-6 compared to robust individuals [9]. In cancer patients, frailty was found to be associated with poor response to chemotherapy, and in patients with operable NSCLC, we previously demonstrated that frailty was associated with various negative postoperative outcomes [10,11].

Blood cell markers of inflammation have recently come to the attention as tools to monitor chronic inflammation and its negative consequences. In patients with resected NSCLC, they have previously been shown to correlate with cancer-negative postoperative outcomes, such as recurrence in lung cancer patients [12]. These biomarkers were also found to correlate with frailty. Easily calculable complete blood count (CBC)-derived biomarkers (such as neutrophil–lymphocyte ratio, NLR; platelet–lymphocyte ratio, PLR; monocyte–lymphocyte ratio, MLR; systemic immune–inflammation Index, SII; and systemic inflammation response index, SIRI) have been shown to correlate with frailty in several populations and diseases [13,14,15]. For example, an extensive retrospective analysis of the National Health and Nutrition Examination Survey (NHANES), involving 13,507 participants, found that all assessed CBC-derived inflammatory biomarkers were associated with frailty status as determined by the Rockwood frailty index [16].

In patients with operable lung cancer, however, the relationship between chronic inflammation and frailty has not been appropriately studied. The present study aims to investigate various “conventional” determinants of frailty, such as age, comorbidity, and nutrition, but focuses on the relationship between blood-derived biomarkers of chronic inflammation and frailty in surgical NSCLC patients.

## 2. Materials and Methods

### 2.1. Study Design

This research is a retrospective study on patients with NSCLC who underwent resection for lung cancer. We investigated all surgical cases of patients with NSCLC from the database of the Respiratory Disease University Hospital, Iasi, Romania, between March 2022 and March 2023.

### 2.2. Cohort

Cases were selected from the database according to whether they underwent lung resection for NSCLC and whether all the necessary data for the calculation of blood-derived biomarkers and frailty scores were available.

### 2.3. Selected Variables

For each case, preoperative demographic data (gender, age, BMI, comorbidity, oncologic history) and CBC values (lymphocytes, platelets, neutrophils, monocytes) were extracted. Based on the selected lab parameters, five blood inflammatory scores were calculated. The NLR, PLR, and MLR were determined by dividing the respective blood count parameters. The SII was calculated by multiplying the platelet and neutrophil counts and dividing the product by the lymphocyte count. The SIRI was calculated by dividing the product of neutrophil and monocyte counts by the lymphocyte count.

### 2.4. Frailty Assessment

Frailty was assessed using two modified frailty indexes based on patient history: mFI-11 and mFI-5, respectively. The methodology for their assessment has been validated in previous studies for thoracic surgery, but their validation in thoracic oncology remains limited [17,18]. The criteria for these comorbidity-derived screening tools are preoperative functional status (dependency level), a history of diabetes mellitus, COPD, current pneumonia, congestive heart failure, high blood pressure in need of treatment, recent myocardial infarction (less than 6 months), a history of myocardial ischemic disease (angina within the last 30 days or history of transcatheter intervention), the presence of altered sensorium, a history of ischemic stroke, the presence of neurologic sequelae following ischemic stroke, the presence of other peristatic vascular diseases (history of revascularization, amputation due to ischemia, pain during gait, gangrene) for mFI-11, and only the first five for mFI-5 (excluding presence of pneumonia). Final scores were calculated by counting all findings for each case. mFI-11 scores ranged from 0 to 11, and a patient was considered frail if at least three criteria were met [17]. The mFI-5 score was assessed similarly, with at least three criteria required to be considered frail and the total score varying from 1 to 5 [18]. In another analysis performed on the same cohort, we demonstrated that mF-5 was able to best predict postoperative outcomes [10].

### 2.5. Statistical Analysis

Statistical analysis was performed using SPSS version 23 (IBM Corp., New York, NY, USA) and focused on two objectives: to evaluate the association between possible predictors of frailty and its presence and severity, and to assess the predictive and discriminative performance of identified predictors. Demographic data were described using descriptive statistics. The normality of continuous data was assessed using the Shapiro–Wilk test. Non-normally distributed variables were reported as medians (interquartile ranges) and compared using the Mann–Whitney U test for two samples or the Kruskal–Wallis test for multiple samples. A further post hoc between-group comparison was performed, and *p* values were adjusted using the Bonferroni correction. Normally distributed variables were reported as the mean (±SD) and compared using the independent-sample t-test and variance analysis using ANOVA. Discrete data were reported as the total count and percentages. Between-group comparisons were performed using the χ^2^ test and the Fisher exact test when appropriate.

To assess the correlation between predictors and frailty severity, Spearman correlation was performed. The strength and direction of the correlation were further evaluated in univariate linear regression. Independent predictors of frailty severity were identified via multivariate linear regression with the forward conditional method to limit model overfitting, given the sample size. This included identified predictors from univariate linear regression. Other covariates included in the multivariate linear model were selected based on exploratory analyses and on known clinical relevance to frailty and oncologic prognosis, such as gender, BMI, stage, and histology. This was performed to reduce any potential residual confounding and was limited to the available data in our database.

To assess the association of selected inflammation predictors and frailty status, we transformed the data using the logarithmic function because the variables were not normally distributed. This was performed to increase further modeling stability and data interpretability [19]. Using the logarithmic values, we analyzed the association of each predictor with frailty status using univariate ANOVA and logistic regression. The discriminative performance of the identified frailty predictors was assessed using ROC (receiver operating characteristic) analysis, with AUC and 95% CI being reported. A significance level of α = 0.05 was considered statistically significant.

### 2.6. Ethics

This project was approved by two institutions and ethics committees, firstly by the Pulmonary Disease University Hospital, Iași, Romania (approval number 105/2023, approval date 10 July 2023), and then by the University of Medicine and Pharmacy “Grigore T. Popa”, Iași, Romania (approval number 416/2024, approval date 15 March 2024). Due to the research’s retrospective design, the requirement for informed consent from patients before data collection was waived. All research was conducted in accordance with the principles of the Declaration of Helsinki.

## 3. Results

The analyzed sample consisted of 106 cases who underwent surgical intervention for NSCLC at the above-mentioned hospital between March 2022 and March 2023 and had available data for frailty and blood cell marker calculations. Of these patients, the majority were male (73.6%) and elderly (62.26% above age 65, mean age 65.4 ± 8.6). Frailty was positively detected in 29.2% of cases when using mFI-11 and in 17% with mFI-5. Sample characteristics are further detailed in Table 1. There was a near-significant difference in frailty scores between genders as identified with mFI-11 (mean score 1.39 ± 1.1 for females versus 2 ± 1.6 for males, *p* = 0.058). However, the difference was significant for mFI-5 scores (mean scores 1.14 ± 0.85 for females versus 1.58 ± 1.11 for males, *p* = 0.02). In this sample, there was a significant difference in comorbidity count between frail and non-frail individuals (*p* < 0.001 for both frailty metrics), while a borderline significant difference was observed for age (*p* = 0.057 for both indexes) and no difference for BMI (*p* = 0.17 for mFI-11 and *p* = 0.61 for mFI-5).

Regarding blood cell biomarkers, the median values in the sample for the NLR, PLR, MLR, SII, and SIRI were 2.45 (1.75–3.49), 132.78 (98.46–173.89), 0.32 (0.25–0.43), 590.54 (402.40–1010.17), and 1.49 (1.03–2.43), respectively. These variables displayed a non-normal distribution. Between-gender comparison revealed a significant difference in MLR and SIRI scores (0.33 (0.27–0.45) for male vs. 0.27 (0.21–0.40) for female for MLR, *p* = 0.02, and 1.65 (1.13–2.66) for male vs. 1.13 (0.75–2.00) for female, *p* = 0.01). However, these scores were also associated with smoking status (*p* = 0.03 for MLR and *p* = 0.004 for SIRI), with the greatest difference observed between non-smokers and former or current smokers in post hoc analysis, which might account for the gender differences observed. No differences in scores were observed for oncologic disease histology and stage, except for the PLR, which was higher in patients with stage IIIB disease (*p* = 0.02). Regarding frailty, the recorded values for the NLR, PLR, and SII were lower in patients with frailty as identified with mFI-5 (1.79 versus 2.67 for NLR, *p* = 0.01, 98.89 vs. 139.76 for PLR, *p* = 0.01, 453.49 vs. 647.41 for SII, *p* = 0.04, for frail and non-frail, respectively), while the observed difference was significant only for the NLR for frailty screened with mFI-11 (2.03 vs. 2.70, *p* = 0.03, for frail and non-frail). When comparing blood count, frail individuals had higher monocyte (0.72 (0.6–1.04) vs. 0.58 (0.47–0.82), *p* = 0.05 for frail and non-frail according to mFI-11 only) and lymphocyte counts (2.33 (1.65–2.82) vs. 1.92 (1.46–2.43), *p* = 0.02 for mFI-11 and 2.49 (1.83–3.30) vs. 1.94 (1.47–2.48), *p* = 0.01 for mFI-5 between frail and non-frail patients). The correlation analysis between CBC scores and sample characteristics is available in Supplementary Table S1.

**Figure 1:**
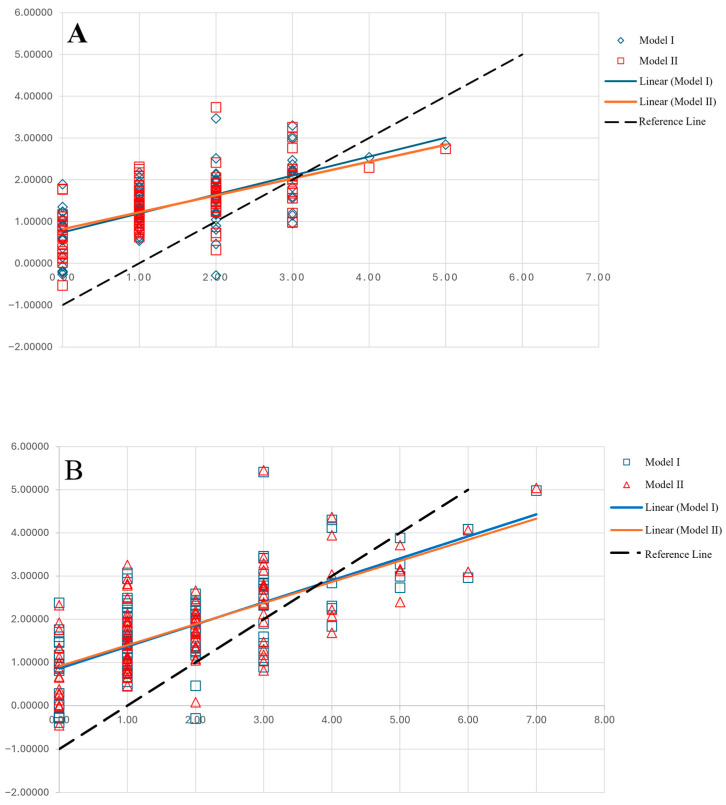
Predictive models for frailty severity using CBC-derived biomarkers. (**A**) Predictive models for mFI-11: **Model I:** mFI-11 = 0.342 X Comorbidity count + 0.049 X Age − 0.243 X NLR + 0.639 X (0 if female/1 if male) − 3.234; **Model II**: mFI-11 = 0.342 X Comorbidity count + 0.046 X Age − 0.004 X PLR + 0.527 X (0 if female/1 if male) − 2.966. (**B**) Predictive models for mFI-5: **Model I**: mFI-5 = 0.190 X Comorbidity count + 0.044 X Age − 0.209 X NLR + 0.435 X (0 if female/1 if male) − 2.399; **Model II:** mFI-5 = 0.187 X Comorbidity count + 0.043 X Age − 0.004 X PLR − 1.615.

Unexpectedly, systemic inflammation scores displayed a modest inverse correlation with frailty burden. This was similar for both frailty scores and the NLR, PLR, and SII (R ≈ −0.2 for all predictors and frailty scores, as detailed in Table 2). Frailty severity was also moderately positively correlated with age (R = 0.35, *p* < 0.001 for mFI-11, and R = 0.36 for mFI-5, *p* < 0.001) and also well correlated with comorbidity burden (R = 0.55, *p* < 0.001 for mFI-11, and R = 5, *p* < 0.001 for mFI-5), while the correlation with nutrition status was borderline significant (R = 0.19, *p* = 0.056 for mFI-11, and R = 0.19, *p* = 0.057 for mFI-5). In linear regression, comorbidity burden, age, the PLR, and the NLR had the largest effects on frailty scores across both screening tools, whereas the SII approached statistical significance (Table 3). This effect was consistent across our multivariate linear regression models, regardless of other covariates, and comorbidity, age, the NLR/PLR, and gender emerged as the best predictors of frailty severity (Table 4). Again, systemic inflammation scores showed an inverse correlation with frailty severity, as in the sample the lower NLR or PLR was associated with higher frailty scores. In these models, male gender was associated with more severe frailty (except for mFI-5 in the PLR model), as reported by the above-mentioned frailty scores. The developed models correlated well with frailty severity (R = 0.7, *p* < 0.001 using NLR, and R = 0.69, *p* < 0.001 using PLR for mFI-11; R = 6.4, *p* < 0.001 using NLR, and R = 0.62, *p* < 0.001 using PLR for mFI-5, respectively), as shown in Figure 1.

**Figure 2:**
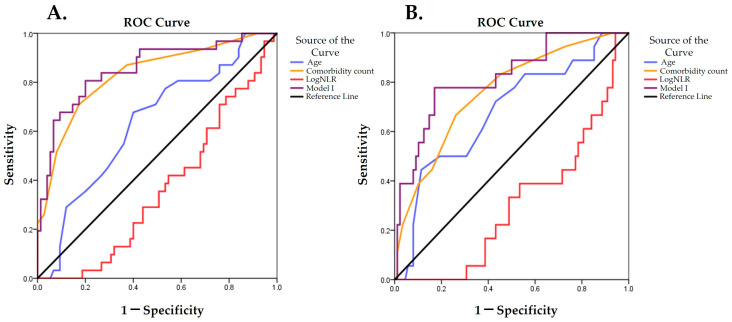
ROC curve of predictors and multivariate logistic model for (**A**) frailty according to mFI-11 and (**B**) frailty according to mFI-5.

In univariate ANOVA, age, comorbidity burden, and logNLR were the best predictors of frailty status for both metrics, while for mFI-5, logPLR was also significant (Table 5). Based on these predictors, we developed two multivariate logistic models, one for both metrics containing age, comorbidity, and logNLR, and one for frailty predicted with mFI-5 only based on age, comorbidity burden, and logPLR. In the first model, all predictors accounted for the change in frailty status. Specifically, a decrease in logNLR was associated with an increased risk of frailty (OR 3.13, 95%CI: 1.12–9.09, *p* = 0.03 for mFI-11, and OR = 3.82, 95%CI:1.28–11.11, *p* = 0.02). In the second model, logPLR was no longer statistically significant for frailty detected with mFI-5 (Table 6). Discriminative performance in ROC analysis was best for comorbidity burden (AUC 0.831, *p* < 0.001), whereas logNLR had poor predictive power (AUC 0.363, *p* = 0.03). However, when all three predictors were included in the model, the first model had the best predictive power for frailty for both metrics (AUC for model 1–0.854, *p* < 0.001, for mFI-11, and AUC-0.821, *p* < 0.001 for mFI-5) (Figure 2). A detailed analysis is reported in Supplementary Table S2.

## 4. Discussion

This study goes further than prior studies in deciphering the predictors of frailty by evaluating the involvement of chronic inflammation using modified frailty indices (mFI-11 and mFI-5) in relation to NSCLC surgery [10]. As demonstrated above, age, comorbidity, and preoperative chronic inflammation are correlated with multidimensional frailty in operable NSCLC patients. Among the available inflammation scores, the NLR was most strongly associated with frailty and inversely correlated with its severity. Lower values of the NLR, PLR, and SII found in frail patients with operable NSCLC indicate the potential role played by chronic inflammation in frailty development [16,20]. In our sample, a decrease in the NLR or PLR was associated with increased frailty probability in a predictable manner. We further show that, despite the overall modest effect of chronic inflammation on frailty relative to age and comorbidity, the NLR/PLR can improve frailty screening models in NSCLC patients, potentially improving outcomes. Furthermore, these markers of inflammation can be modified to a greater extent compared to comorbidities (partially modifiable) or with age (non-modifiable). This has been shown recently in other surgical cohorts where preoperative immune prehabilitation has been found to improve postoperative outcomes [21]. Given that frailty is partially reversible, identifying frail lung cancer surgical patients with chronic inflammation via these scores might be an opportunity to select patients for immune prehabilitation and frailty preoperative optimization, probably improving surgical suitability and outcomes [22].

For lung cancer patients, elevated systemic inflammation has been reported and associated with unfavorable patient evolution. In surgical patients with stage I NSCLC, cutoff values for the NLR, SII, and SIRI were identified to be 2.6, 580.7, and 0.71, respectively, and exceeding these values was associated with poorer outcomes [23]. The NLR was also associated with decreased survival in surgical lung cancer patients with advanced stages of disease (IIIA and IIIB) [24]. The NLR reflects the immune setup within the tumor microenvironment [25]. This association among individuals with lung cancer can be explained by two mechanisms. One is the increased neutrophil levels due to tumor growth and disease progression, and the other is the decreased lymphocyte levels associated with immunosuppression in patients with cancer [25,26]. This increase in neutrophil/trombocite levels and the decrease in lymphocyte levels reflect an acute systemic immune response rather than chronic inflammation. Across the entire sample in our study, the NLR, SII, and SIRI were elevated and approached the cutoff values reported in the literature for surgical patients with lung cancer (Supplementary Table S1). However, our observed values of the CBC-derived biomarkers were significantly lower in frail individuals. For instance, the NLR, PLR, and SII levels in this study were 1.79, 98.89, and 453.49, respectively (for mFI-5), which are comparable to the normal values observed in the elderly general population, as reported by Fest et al. (NLR: 1.76 (0.83–3.92), PLR: 120 (61–239), and SII: 459 (189-1168)) [27]. Moreover, this association was linear; with each decrease in the NLR, frailty severity increased. This suggests that in our sample, in contrast to the observed and expected trends in inflammatory response in lung cancer patients, frail individuals manifest an immune response shift. In our data, this was mostly due to an increase in lymphocyte counts rather than a decrease in neutrophils, supporting the association with chronic inflammation.

Frailty was determined in this case using two comorbidity-based indices, and thus, the strong correlation with comorbidity was expected. Given that some comorbidities used in the assessment are strongly correlated with systemic inflammation, such as COPD and diabetes mellitus, the association between frailty and the increased NLR/PLR is expected [28]. This inverse relationship is counterintuitive, as other frail cohorts have been shown to have elevated NLR/PLR levels [16]. In our lung cancer surgical patient sample, this might also be explained by the immune exhaustion or immune redistribution often seen in lung cancer patients, and thus might represent a particularity only for frail lung cancer patients [29,30]. In our study, the trend observed between CBC inflammation scores and frailty is probably explained by immune senescence. Studies show that frail individuals exhibit increased monocyte activation and reduced mononuclear responses in vitro due to desensitization and immune senescence [31]. We note that the discrimination of inflammatory scores for frailty in the ROC curve was poor and not sufficient as a stand-alone predictor of frailty status. To determine whether chronic inflammation is a determinant of frailty in lung cancer patients, further prospective analyses are required and frailty should be assessed using metrics beyond comorbidity-based instruments, such as the Fried phenotype.

Other previous studies evaluated these blood markers as predictors of disease outcome in patients with lung cancer. One such study involved an index that we were not able to use due to the peculiarities of cohort: the prognostic nutritional index, which involves both albumin and lymphocyte count, was found to predict overall survival in a large cohort of approximately 700 small-cell lung cancer patients [32]. When this index was combined with the NLR in a cohort of 334 patients who underwent curative surgery for NSCLC, both indices were found to be independent predictors of survival [33]. These markers were also associated with sex, smoking status, tumor size, and vascular invasion. In a cohort of 311 patients with advanced NSCLC treated with chemotherapy or with therapies targeting growth factors, the systemic immune inflammation index was found to predict rapid progression [34]. However, few studies examined the relationship between inflammation, blood biomarkers, and frailty. For instance, two studies evaluated frailty in the general population of the United States using the NHANES database. The first investigated patient data between 1999 and 2016 [16]. They also used logarithmic normalized values of CBC biomarkers and found increased logNLR, logMLR, logSII, and logSIRI associated with frailty presence (OR: 3.45, *p* < 0.001; 3.58, *p* < 0.001; 1.89, *p* < 0.001; and 2.77, *p* < 0.001). However, frailty was assessed using the cumulative deficit frailty tool (Rockwood frailty tool), and the correlation displayed a non-linear pattern with frailty scores. In a further analysis performed on the same database for the period 1999 to 2018 using a 36-item frailty index, raw NLR, PLR, MLR, SII, and SIRI values were associated with increased frailty risk (OR = 1.96, OR = 1.13, OR = 1.81, OR = 1.56, and OR = 2.03, respectively; *p* < 0.001 for each) [35]. This has been confirmed in other populations as well. In an inpatient geriatric sample from a university hospital in China, inflammation was positively correlated with frailty, as assessed by a five-item FRAIL scale (r = 0.12, *p* = 0.004) [36]. However, their sample, which was also a smaller cohort, consisted predominantly of frail and prefrail patients (49.38% and 34.33%, respectively) who were admitted for decompensation of chronic conditions, and thus, bias related to morbid and comorbid burdens might have been probable.

Our study has as items of originality the in-depth analysis of frailty predictors, including blood cell inflammation markers, as well as the target population of operable NSCLC. The fact that we found differences in NLR, PLR, and SII scores between frail and non-frail individuals (only for NLR with mFI-11), and that these scores correlate with frailty in an inverse-proportional manner, suggests that chronic inflammation might be involved in frailty development. Given that the frailty indices used are mostly based on comorbidity burden, these results are not unusual, as many of them involve chronic inflammation as a pathogenic mechanism. Another hypothesis might be represented by inflammaging (chronic inflammation associated with aging), but to demonstrate it more accurately, we would have needed elderly patients with operable NSCLC and no inflammatory comorbidities. Even in this case, cancer-related inflammation would have been very difficult to distinguish from inflammaging, because the blood cell biomarkers used are not specific. Furthermore, certain biomarkers, such as the SIRI, can reflect both acute and chronic inflammation, and the fact that we did not find any correlation between this marker and frailty is not unusual—in fact, the contrary [16].

Therefore, such blood cell biomarkers can help predict survival, frailty, and, in the case of operable NSCLC, postoperative outcome. It would be interesting to determine whether these blood cell biomarkers can be modified by treatment, thereby improving the aforementioned outcomes.

This study has several limitations. We did not perform a sample size calculation; therefore, no data are available on sample appropriateness. Our study analyzed a small sample of surgical lung cancer cases, which limits the generalizability of these findings. The retrospective design is another limitation of this study. We evaluated CBC biomarkers and frailty at a single time point before the surgical intervention; thus, in the absence of follow-up, a causal association between frailty and inflammatory status is not possible. It remains unclear whether frailty causes the observed differences in immune response or the resultant effects. Moreover, the small incremental effect observed for inflammation might be due to the comorbidity burden used in the frailty score calculation. Given the circular relationship between frailty scores and the burden of comorbidities, this must be carefully considered. This was a single-center study and may not be representative of lung cancer patients elsewhere. Furthermore, the data collected depend on accurate medical record documentation and are subject to bias.

## 5. Conclusions

Our study found that frailty among lung cancer patients is associated with age, comorbidity burden, and inflammation. The NLR was identified as the best CBC inflammation score correlated with frailty status and severity. Interestingly, frail individuals exhibited lower NLR levels, suggesting a potential shift in immune response mechanisms distinct from the typical systemic inflammation increase seen in cancer progression. Further studies are required to understand the causal relationship between frailty and inflammation, and to explore their implications for personalized management in operable NSCLC.

## Figures and Tables

**Table 1:** Sample characteristics.

Sample Characteristics	
Age Mean (±SD)	65.4 (±8.6)
Gender	
Male No. (%)	78 (73.6%)
Female No. (%)	28 (26.4%)
BMI Mean (±SD)	26.9 (±4.59)
Comorbidities No. (%)	4 (±2)
Smoking exposure	
Active Smoker No. (%)	38 (35.8%)
Stopped Smoking No. (%)	28 (26.4%)
Never Smoked No. (%)	36 (34%)
Histology	
Adenocarcinoma No. (%)	70 (66%)
Squamous cell carcinoma No. (%)	26 (24.5%)
Other carcinoma types No. (%)	6 (5.7%)
Undifferentiated carcinoma No. (%)	4 (3.8%)
Cancer stage	
IA No. (%)	18 (17%)
IB No. (%)	20 (18.9%)
IIA No. (%)	9 (8.5%)
IIB No. (%)	18 (17%)
IIIA No. (%)	9 (8.5%)
IIIB No. (%)	8 (7.5%)
IV No. (%)	24 (22.6%)
Frailty prevalence	
mFI-11	
Frail No. (%)	31 (29.2%)
Non-Frail No. (%)	75 (70.8%)
mFI-5	
Frail No. (%)	18 (17%)
Non-Frail No. (%)	88 (83%)
Total No. (%)	106 (100%)

**Table 2:** Correlation analysis between blood inflammation biomarkers and frailty scores.

CBC Biomarkers	mFI5		mFI11	
	R	*p*	R	*p*
NLR	−0.22	0.02	−0.22	0.03
PLR	−0.23	0.02	−0.25	0.01
MLR	−0.11	0.28	−0.10	0.32
SII	−0.2	0.04	−0.21	0.03
SIRI	−0.08	0.45	−0.05	0.60

**Table 3:** Univariate linear regression between frailty scores and blood inflammation biomarkers, age, and comorbidity burden.

Variable	mFI-11 Score				mFI-5 Score			
	Coefficient (B)	Standardized Coefficient (Beta)	95%CI for B	*p*	Coefficient (B)	Standardized Coefficient (Beta)	95%CI for B	*p*
Age	0.056	0.316	0.023 to 0.088	0.001	0.045	0.36	0.022 to 0.067	<0.001
Comorbidity count	0.383	0.61	0.286 to 0.48	<0.001	0.223	0.507	0.149 to 0.297	<0.001
NLR	−0.198	−0.218	−0.371 to −0.025	0.025	−0.144	−0.226	−0.264 to −0.023	0.02
PLR	−0.006	−0.254	−0.01 to −0.001	0.009	−0.004	−0.258	−0.007 to −0.001	0.008
MLR	−0.881	−0.095	−2.685 to 0.923	0.335	−0.838	−0.129	−2.094 to 0.418	0.189
SII	−0.000423	−0.19	−0.001 to 0.000002	0.051	−0.000423	−0.185	−0.001 to 0.000002	0.057
SIRI	−0.076	−0.098	−0.227 to 0.074	0.316	−0.056	−0.104	−0.161 to 0.049	0.291

**Table 4:** Multivariate linear regression models that best predict frailty severity.

	mFI-11 Score				mFI-5 Score			
	Coefficient (B)	Standardized Coefficient (Beta)	95%CI for B	*p*	Coefficient (B)	Standardized Coefficient (Beta)	95%CI for B	*p*
Models for NLR								
NLR	−0.243	−0.231	−0.396 to −0.091	0.002	−0.209	−0.289	−0.316 to −0.101	<0.001
Age	0.049	0.268	0.022 to 0.075	<0.001	0.044	0.35	0.025 to 0.062	<0.001
Gender	0.639	0.184	0.146 to 1.132	0.012	0.435	0.182	0.086 to 0.784	0.015
Comorbidity burden	0.342	0.544	0.251 to 0.434	<0.001	0.19	0.439	0.125 to 0.254	<0.001
Models for PLR								
PLR	−0.004	−0.171	−0.008 to −0.001	0.025	−0.004	−0.232	−0.006 to −0.001	0.004
Age	0.046	0.254	0.019 to 0.073	0.001	0.043	0.342	0.023 to 0.062	<0.001
Gender	0.527	0.151	0.02 to 1.035	0.042				
Comorbidity burden	0.342	0.545	0.248 to 0.437	<0.001	0.187	0.434	0.119 to 0.256	<0.001

**Table 5:** Univariate ANOVA analysis of predictors of frailty status.

	Frail According to mFI-11			Frail According to mFI-5		
	F-Statistic	Effect Size	*p*	F-Statistic	Effect Size	*p*
Age	3.72	0.76	0.05	3.71	0.52	0.05
Comorbidity	45.03	6.63	<0.001	13.98	1.77	<0.001
BMI	1.92	0.40	0.17	0.26	0.04	0.61
logNLR	4.32	0.88	0.04	4.87	0.67	0.03
logPLR	2.44	0.50	0.12	4.26	0.59	0.04
logMLR	0.53	0.11	0.47	0.39	0.06	0.53
logSII	1.56	0.32	0.22	3.34	0.46	0.07
logSIRI	0.33	0.07	0.57	0.52	0.07	0.47

**Table 6:** Multivariate logistic regression models for prediction of frailty status.

		Frailty According to mFI-11		Frailty According to mFI-5	
		OR (95%CI)	*p*	OR (95%CI)	*p*
Model I	AGE	1.071 (0.99–1.16)	0.09	1.104 (1.003–1.22)	0.04
	Comorbidity	1.952 (1.443–2.64)	<0.001	1.37 (1.09–1.72)	0.006
	LogNLR	0.319 (0.11–0.89)	0.03	0.261 (0.09–0.78)	0.02
Model II	AGE			1.08 (0.99–1.19)	0.08
	Comorbidity			1.36 (1.08–1.69)	0.01
	LogPLR			0.37 (0.13–1.09)	0.07

## Data Availability

The data presented in this study are available on request from the corresponding author (data are not publicly available due to ethical approval restrictions).

## Supplementary Materials

The following supporting information can be downloaded at https://www.mdpi.com/article/10.3390/medsci14020170/s1: Table S1: Correlation between demographic characteristics and CBC inflammation scores; Table S2: ROC curve analysis for the best predictors of frailty.

## Abbreviations

The following abbreviations are used in this manuscript:

CRP	C-reactive protein
CBC	Complete Blood Count
NLR	Neutrophil–Lymphocyte Ratio
PLR	Platelet–Lymphocyte Ratio
MLR	Monocyte–Lymphocyte Ratio
SII	Systemic Immune–Inflammation Index
SIRI	Systemic Inflammation Response Index
mFI-11	Modified Frailty Index with 11 items
mFI-5	Modified Frailty Index with 5 items
NHANES	National Health and Nutrition Examination Survey
BMI	Body Mass Index
NSCLC	Non-Small-Cell Lung Cancer
COPD	Chronic Obstructive Pulmonary Disease
