# Supplementary material for: The Association Between Inflammatory Scores and Frailty Severity: An Exploratory Retrospective Analysis in Non-Small-Cell Lung Cancer Surgical Patients
Source: Med Sci (Basel). 2026 Mar 28;14(2):170. doi: 10.3390/medsci14020170 (PMC13108116; doi:10.3390/medsci14020170)
Supplement: Supplementary file 1 [file medsci-14-00170-s001.zip › medsci-4112036-supplementary.pdf]

Supplementary Table S1. Correlation between demographic characteristics and CBC inflammation scores.

	NLR Median (IQR)	P	PLR Median (IQR)	P	MLR Median (IQR)	P	SII Median (IQR)	P	SIRI Median (IQR)	P
**Age**	0.18*	0.07	0.12*	0.23	0.16*	0.10	0.08*	0.44	0.13*	0.18
**Gender**		0.53**		0.42**		0.02**		0.62**		0.01**
Male	2.67(1.73 -3.65)		132.74(90.65-175.07)		0.33(0.27 -0.45)		636.08(402.40-1042.88)		1.65(1.13 -2.66)	
Female	2.24(1.77 -3.23)		133.17(113.10 -162.52)		0.27(0.21 -0.40)		529.07(397.58-934.01)		1.13(0.75 -2.00)	
**BMI**	-0.13*	0.18	-0.17*	0.09	-0.13*	0.19	-0.19*	0.06	-0.16*	0.10
**Comorbidities**	-0.13*	0.18	-0.12*	0.22	-0.11*	0.25	-0.10*	0.30	-0.06*	0.54
**Smoking exposure**		0.43***		0.93***		0.03***		0.26***		0.004***
Active Smoker	2.56(1.44 -3.81)		127.05(90.17-180.05)		0.32(0.25 -0.45)		760.71(355.22-1260.34)		1.70(1.10 -3.36)	
Stopped Smoking	2.72(1.95 -3.59)		140.72(92.23-175.22)		0.37(0.30 -0.48)		687.71(447.71-860.27)		2.19(1.27 -2.50)	
Never Smoked	2.24(1.76 -3.12)		132.78(111.97 -156.63)		0.29(0.21 -0.40)		509.73(395.36-805.73)		1.18(0.74 -1.62)	
**Histology**		0.23***		0.24***		0.58***		0.54***		0.70***
Adenocarcinoma	2.22(1.63 -3.40)		127.65(100.33 -159.21)		0.31(0.23 -0.43)		579.54(386.79-1010.17)		1.37(0.83 -2.53)	
Squamous cell carcinoma	2.53(2.11 -3.06)		140.41(87.04-176.38)		0.34(0.28 -0.41)		613.35(459.50-903.32)		1.70(1.25 -2.28)	
Other carcinoma types	2.87(1.72 -4.51)		136.93(103.64 -220.89)		0.40(0.22 -0.54)		629.50(337.10-1468.56)		1.69(0.92 -3.48)	
Undifferentiated carcinoma	3.97(2.63 -6.22)		218.11(134.67 -313.04)		0.40(0.32 -0.42)		1199.69(594.85 -1545.23)		1.97(1.23 -2.56)	
**Cancer stage**		0.26***		0.02***		0.25***		0.23***		0.57***
IA	1.82(1.61 -2.56)		109.94(74.36-127.60)		0.26(0.22 -0.33)		480.37(369.14-684.57)		1.18(0.91 -2.22)	
IB	2.35(1.96 -2.97)		120.81(96.02-148.90)		0.31(0.24 -0.44)		547.09(389.60-805.17)		1.46(0.88 -2.22)	
IIA	2.68(2.18 -2.95)		146.89(125.30 -161.14)		0.31(0.29 -0.44)		646.32(571.39-1085.58)		1.53(1.24 -2.71)	
IIB	2.66(2.18 -3.58)		152.39(116.96 -188.09)		0.36(0.27 -0.56)		678.16(429.69-1120.82)		1.81(1.15 -3.36)	
IIIA	2.16(1.37 -3.71)		102.82(84.21-163.51)		0.33(0.22 -0.41)		388.94(315.18-1271.37)		1.66(0.76 -2.69)	
IIIB	3.25(1.97 -4.18)		214.30(113.15 -229.41)		0.40(0.24 -0.53)		1112.09(472.83 -1390.39)		2.31(1.14 -3.16)	
IV	2.97(1.52 -4.34)		140.57(94.82-201.94)		0.33(0.24 -0.44)		764.78(374.64-1230.20)		1.59(0.84 -2.53)	
**Frailty prevalence**										

mFI-11		0.03**		0.11**		0.29**		0.17**		0.71**
Frail	2.03(1.62-2.85)		123.01(82.83-165.10)		0.29(0.25-0.37)		532.00(396.85-818.8)		1.35(1.12-2.38)	
Non-Frail	2.70(1.84-3.75)		139.07(105.59-179.66)		0.33(0.25-0.43)		646.32(419.46-1172.67)		1.63(0.93-)	
mFI-5		0.01**		0.01**		0.35**		0.04**		0.55**
Frail	1.79(1.35-2.72)		98.89(79.30-133.27)		0.27(0.24-0.40)		453.49(355.22-769.25)		1.31(1.09-2.29)	
Non-Frail	2.67(1.88-3.72)		139.76(107.06-176.24)		0.33(0.25-0.43)		647.41(419.71-1166.63)		1.54(1.03-2.46)	
**Total**	2.45(1.75-3.49)		132.78(98.46-173.89)		0.32(0.25-0.43)		590.54(402.40-1010.17)		1.49(1.03-2.43)	

* Spearman R; **Mann–Whitney U test;***Kruskal–Wallis test.

Supplementary Table S2. ROC curve analysis for the best predictors of frailty.

	mFI-11			mFI-5		
	AUC	95%CI	P	AUC	95%CI	P
Age	0.634	0.52-0.748	0.03	0.678	0.54-0.816	0.02
Comorbidity	0.831	0.742-0.92	<0.001	0.759	0.64-0.879	0.001
LogNLR	0.363	0.255-0.471	0.03	0.306	0.185-0.427	0.01
Model I	0.854	0.77-0.938	<0.001	0.821	0.713-0.929	<0.001
